# Maintenance of body weight is an important determinant for the risk of ischemic stroke: A nationwide population-based cohort study

**DOI:** 10.1371/journal.pone.0210153

**Published:** 2019-01-03

**Authors:** Jung-Hwan Cho, Eun-Jung Rhee, Se Eun Park, Hyemi Kwon, Jin-Hyung Jung, Kyung-Do Han, Yong-Gyu Park, Soon-Jib Yoo, Yang-Hyun Kim, Won-Young Lee, Taskforce Team of the Obesity Fact Sheet of the Korean Society for the Study of Obesity

**Affiliations:** 1 Division of Endocrinology and Metabolism, Department of Internal Medicine, Kangbuk Samsung Hospital, Seoul, Republic of Korea; 2 Department of Medical Statistics, Catholic University College of Medicine, Seoul, Republic of Korea; 3 Division of Endocrinology and Metabolism, Department of Internal Medicine, Bucheon St. Mary's Hospital, College of Medicine, The Catholic University of Korea, Bucheon, Republic of Korea; 4 Department of Family Medicine, Korea University Hospital, College of Medicine, Korea University, Seoul, Republic of Korea; University of Zurich, SWITZERLAND

## Abstract

**Background:**

Overweight is known as a risk factor for ischemic stroke. However, the effect of weight change on the development of ischemic stroke remains controversial. We investigated the relationship between weight change and the risk of ischemic stroke using a nationwide population-based cohort.

**Methods:**

Our study enrolled 11,084,683 participants (Mean age 49.7±13.5 years, range 20–114 years) in the Korean National Health Screening Program from 2009 to 2012. Weight change was calculated using the difference between the baseline weight and the weight at health screening four years prior to the baseline. The occurrence of newly-diagnosed ischemic stroke was observed until the end of 2015. We categorized the study population according to weight change and performed multivariable analysis to compare the risk.

**Results:**

Ischemic stroke was newly diagnosed in 113,591 subjects. The crude incidence rates of ischemic stroke per 1000 person-years according to the change in body weight were 3.059, 1.906, and 1.491 in the <-5%, ±5%, and ≥+5% groups, respectively. After adjusting all variables, the hazard ratio (HR) of ischemic stroke was higher in subjects who underwent weight loss (HR 1.152) or weight gain (HR 1.087) than in those who maintained their weight. When analyzed by eight groups of 5% intervals, the risk showed a U-shaped curve with those who maintained their weight showing the lowest risk.

**Conclusions:**

The risk of ischemic stroke was gradually increased in those who lost or gained more than 5% of their weight over four years, after adjusting for confounders. We should be aware of the increased risk of ischemic stroke in people who undergo weight change and should identify and manage the cause of weight change.

## Introduction

Obesity is steadily increasing worldwide and is a serious problem associated with approximately 3.5 million deaths [1]. In 2013, 10.3 million cases of stroke occurred, of which two-thirds were ischemic strokes [2].

The association between overweight and the risk of ischemic stroke is well known [3], and overweight and obesity are independently associated with a gradual increase in ischemic stroke risk [4]. In addition, the major risk factors for stroke are also closely linked to obesity [5]. Thus, the recent guideline published by the American Stroke Association recommends weight reduction for overweight or obese individuals with a body mass index (BMI) equal to 25 kg/m^2^ or greater for the primary prevention of ischemic stroke [6].

However, the effect of weight change on ischemic stroke risk remains controversial. Weight gain is known to increase the risk of ischemic stroke [7], and weight loss, through maintaining a negative energy balance, reduces the risk [8,9]. However, in an observational study based on the Framingham study participants, cardiovascular mortality was increased for both men and women who lost weight compared to those who maintained their weight [10]. Other studies on middle-aged participants also showed higher cardiovascular mortality in the case of losing weight [11,12]. Furthermore, studies have shown an increased risk of ischemic stroke in weight loss [13,14]. However, some controversies remain regarding whether weight gain or loss could affect the occurrence of ischemic stroke.

Therefore, we investigated the relationship between weight change and the incidence and risk of ischemic stroke to clarify its relevance by using the Korean Nationwide Health Screening Database. We hypothesized that the risk of ischemic stroke is greater for those who gained or lost weight than for those who maintained their weight.

## Methods

### Korean nationwide health screening database

The National Health Insurance Service (NHIS) organized by the Korean government provides health insurance services for more than 97% of Koreans. The demographic information of beneficiaries such as disease codes, medical examinations, and claims for treatment are secured by the NHIS. Beneficiaries of the NHIS older than 40 years and insured workers older than 20 years undergo annual or biennial health screening supported by the NHIS. The health screening database includes information on health behaviors, self-questionnaires, anthropometric measurements, and laboratory findings; the demographic information of beneficiaries was combined to constitute a nationwide population-based cohort [15]. All researchers had access to the database without restrictions.

This study was approved by the Institutional Review Board of the Kangbuk Samsung Hospital (approval number: KBS12089), and authorized by the NHIS (research number NHIS-2017-1-201). All data were fully anonymized before we accessed them and IRB of Kangbuk Samsung Hospital waived the requirement for informed consent.

### Study population

Our study involved 23,503,802 Koreans aged ≥20 years who underwent the health screening more than once, from 2009 to 2012. If the participants received multiple health screenings during that period, the first health checkup was set as the baseline.

**Fig 1:**
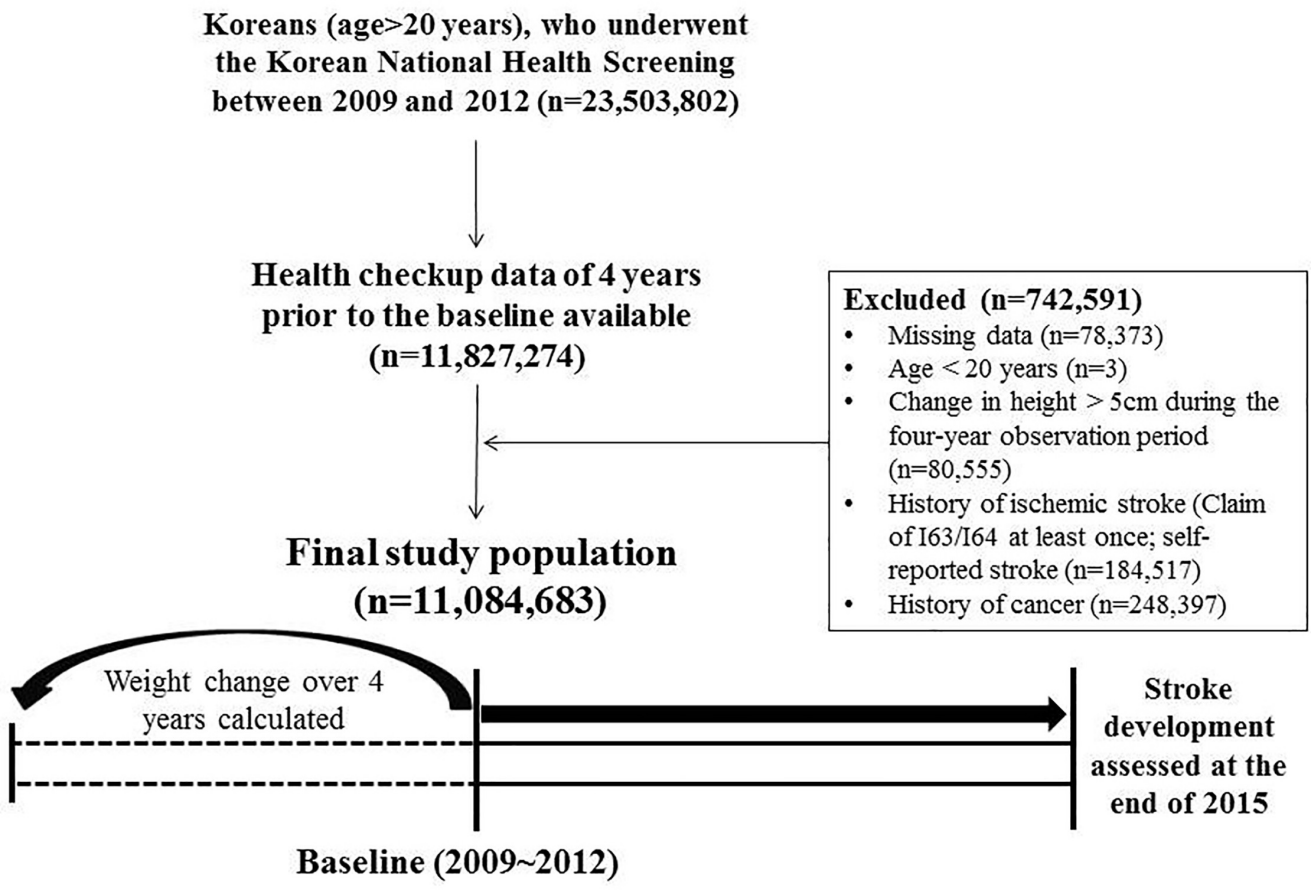
Flowchart of selection of the final study population.

We defined weight change as the difference between the baseline weight of the participants and their weight four years earlier by checking health screening records four years prior to the baseline. Therefore, participants without a health screening record from four years prior to the baseline were not eligible for the study (n = 11,827,274) (Fig 1). Participants with missing data of baseline characteristics or covariates (n = 78,373), those <20 years of age (n = 3), and those with a change in height >5 cm during the four-year observation period (n = 80,555) were also excluded. Participants who were diagnosed with ischemic stroke according to the 10^th^ International Statistical Classification of Diseases and Related Health Problems (ICD-10), code I63 or I64 more than once, and those with a history of stroke as identified in the self-questionnaire (n = 184,517) were further excluded. In addition, people who had a history of cancer (n = 248,397) were also excluded because cancer is a key cause of unintentional weight loss. Finally, 11,084,683 participants were enrolled in our study (Fig 1).

### Baseline assessments

Basic information and health-related behaviors, including smoking (never or current), drinking alcohol (none or heavy; more than 30 g per day), and regular physical activity (vigorous exercise more than three days per week or moderate exercise more than five days per week), were collected with standardized health screening questionnaires. All data of BMI and body weight were measured by trained examiners. Blood pressure was measured using a blood pressure monitor after five minutes of rest, and all blood tests were performed after eight hours of overnight fasting.

Diabetes was defined as a fasting blood glucose level ≥126 mg/dL (≥7 mmol/L) or the presence of one or more claims per year for anti-hyperglycemic medications with ICD-10 code E10-14. Hypertension was defined as systolic blood pressure ≥140 mmHg and diastolic blood pressure ≥90 mmHg or the presence of one or more claims per year for anti-hypertensive medications with ICD-10 code I10-I15. Dyslipidemia was defined as total cholesterol levels ≥240 mg/dL (≥6.21 mmol/L) according to the 2015 Korean Guidelines for the Management of Dyslipidemia or the presence of one or more claims per year for anti-hyperlipidemic medications with ICD-10 code E78 [16].

### Study design and outcomes

We defined the weight maintenance group as weight change within ±5% and categorized the study participants into three and eight groups according to weight change by 5% intervals [11,12].

The primary endpoint was the occurrence of first ischemic stroke, defined as the event-related hospitalization along with the ICD-10 code I63 or I64 and the presence of a claim for computed tomography or magnetic resonance imaging. This outcome was observed by checking the NHIS database until the end of 2015.

In addition, we stratified the BMI at baseline and the BMI four years prior to the baseline into five levels according to the Clinical Practice Guidelines and the Korean Society for the Study of Obesity [17] and investigated how the risk of stroke differed according to the changes in BMI over four years: BMI level 1, BMI <18.5 kg/m^2^; BMI level 2, 18.5≤ BMI < 3.0 kg/m^2^; BMI level 3, 23.0≤ BMI <25.0 kg/m^2^; BMI level 4, 25.0≤ BMI <30.0 kg/m^2^; BMI level 5, BMI ≥30.0 kg/m^2^.

### Statistical analysis

Analysis of variance was used to analyze the data of baseline characteristics presented as continuous variables, while chi-square tests were used for the analysis of categorical variables. The incidence rate (IR) was defined as the prevalence of the outcome per 1000 person-years. If follow-up was lost due to death, the period of observation until that time was included in the total follow-up duration. The risk of ischemic stroke according to weight change was analyzed using Cox regression to evaluate hazard ratios (HRs) with 95% confidence intervals (CIs). We compared the HRs of weight change groups using the group that maintained initial weight as a reference. To identify independent relationships, we performed further adjustments including variables that were associated with weight change and ischemic stroke. The confounding variables to be included in multivariate analysis were the variables that affected the outcome of the model. As the size of the total study population was huge, all variables showed significant differences according to the presence or absence of the outcome. Therefore, we selected the variables considering clinical significance and multicollinearity. The HRs for ischemic stroke in subgroups divided according to various risk factors related with weight change were analyzed by Cox regression analyses with P for interaction between the subgroups. Diabetes, hypertension and dyslipidemia were categorized as the mediators, not confounding variables. We performed subgroup analyses according to the presence or absence of these mediators, instead of including them in the model.

All statistical analyses were performed using SAS version 9.3 (SAS Institute Inc., Cary, NC, USA). A P-value less than 0.05 was considered statistically significant.

## Results

### Baseline characteristics of the participants

Of the 11,084,683 participants (mean age 49.7±13.5 years, range 20–114), 1,528,869 people lost more than 5% of their weight, 7,341,936 people maintained their weight within ±5% of the initial measurement, and 2,213,878 people gained weight by more than 5% (Table 1). The weight loss group had a higher proportion of women, participants aged ≥60 years, non-smokers, regular physical activity, low income, no abdominal obesity, pre-BMI >25 kg/m^2^, and a higher prevalence of diabetes, hypertension, dyslipidemia, ischemic heart disease, chronic obstructive pulmonary disease, and chronic kidney disease than the weight gain group.

**Table 1:** Baseline characteristics of participants according to changes in weight.

Characteristics	Change in weight		
	<-5% (n = 1,528,869)	±5% (n = 7,341,936)	>+5% (n = 2,213,878)
Age (years)	53.2±14.5	50.4±13	45±13.5
Age ≥60 years (%)	544,028 (35.6)	1,904,085 (25.9)	367,644 (16.6)
Male (%)	736,584 (48.2)	4,113,154 (56.0)	1,246,420 (56.3)
Height (cm)	161.7±9.3	163.7±9.1	164.9±9.3
Weight (kg)	59.3±10.7	64.1±11.1	67.5±12.1
BMI (kg/m^2^)	22.6±3.0	23.8±3.0	24.7±3.3
Waist circumference (cm)	78.2±8.7	80.8±8.8	82.2±9.0
Fasting glucose (mg/dL)	100.5±30.1	97.7±21.4	95.8±18.4
Systolic BP (mmHg)	122.3±15.3	122.8±14.8	122.5±14.5
Diastolic BP (mmHg)	75.8±9.9	76.5±9.9	76.6±9.8
Total cholesterol (mg/dL)	192.7±36.9	196.9±36.3	198.4±36.7
Triglyceride (mg/dL)	103.36 (103.27–103.45)	116.11 (116.06–116.16)	123.07 (122.97–123.16)
HDL-C (mg/dL)	56.6±18.8	54.9±18.4	54.4±17.8
LDL-C (mg/dL)	113.1±42.8	116.2±42.8	116.7±44.8
GFR (mL/min/1.73 m^2^)	87.6±36.2	87.3±38.9	89.6±41
Current smoker (%)	335,023 (21.91)	1,712,054 (23.32)	567,147 (25.62)
Heavy drinker (%)	98,122 (6.42)	553,510 (7.54)	176,778 (7.98)
Doing regular physical activity (%)	328,379 (21.48)	1,480,134 (20.16)	357,673 (16.16)
Low income (lowest 20%) (%)	285,225 (18.66)	1,168,900 (15.92)	337,931 (15.26)
Pre-BMI >25 (%)	640,843 (41.92)	2,385,427 (32.49)	446,683 (20.18)
Post-BMI >25 (%)	294,954 (19.29)	2,434,674 (33.16)	950,133 (42.92)
Abdominal obesity (%)	338,469 (22.14)	2,087,647 (28.43)	747,600 (33.77)
Diabetes (%)	223,995 (14.65)	686,533 (9.35)	138,378 (6.25)
Hypertension (%)	484,962 (31.72)	2,100,330 (28.61)	515,544 (23.29)
Dyslipidemia (%)	325,480 (21.29)	1,565,093 (21.32)	435,780 (19.68)
IHD (%)	101,846 (6.66)	375,997 (5.12)	82,596 (3.73)
COPD (%)	116,338 (7.61)	449,482 (6.12)	118,236 (5.34)
CKD (%)*	112,953 (7.39)	453,299 (6.17)	116,542 (5.26)
End stage renal disease (%)	1,875 (0.12)	3,363 (0.05)	1,073 (0.05)
Duration (years)	5.1±1.2	5.2±1.2	5.1±1.2

All data met P <0.0001. Data are presented as means±SD, geometric means (95% confidence intervals), or frequency (%). BMI, body mass index; IHD, ischemic heart disease; COPD, chronic obstructive pulmonary disease; CKD, chronic kidney disease; BP, blood pressure; HDL-C, high-density lipoprotein cholesterol; LDL-C, low-density lipoprotein cholesterol; GFR, glomerular filtration rate; SD, standard deviation

* Defined as a decline in estimated GFR less than 60 mL/min/1.73 m^2^

### Incidence and HRs of ischemic stroke

From 2009 to the end of 2015, 113,591 participants (approximately 1.02% of the total) were newly diagnosed with ischemic stroke. The mean follow-up period of participants was 5.18±1.18 years, and the median time to ischemic stroke development was 2.81 years.

The crude IRs of ischemic stroke per 1000 person-years was 3.06 (<-5%), 1.91 (±5%), and 1.49 (≥+5%) for the entire population (Table 2). A larger adjusted IR (adjusted for age and sex) and HR after adjusting for age and sex (Model 1) were confirmed for the weight gain group (adjusted IR 0.96; HR 1.10, 95% CI 1.08–1.12) than for the weight maintenance group (adjusted IR 0.87; HR 1.000, reference), contrary to the results of the crude IR. After adjusted analyses considering all variables (Model 3), the HRs of ischemic stroke were higher in participants who underwent weight loss (<-5%) (HR 1.21, 95% CI 1.19–1.22) or weight gain (≥+5%) (HR 1.06, 95% CI 1.05–1.08) than in those who maintained their weight. When these analyses were performed in subgroups of mediators, that is, presence or absence of diabetes, hypertension or dyslipidemia, similar results were observed, and all mediators showed interaction with the association between weight change and ischemic stroke (S1 Table).

**Table 2:** Incidence rate and multivariable adjusted HRs (95% CIs) of ischemic stroke (in three groups).

Subgroups	Frequency	Number of events	IRs (per 1,000 person-years)	Multivariable-adjusted HRs (95% CI)		
				Model 1	Model 2	Model 3
Total						
<-5%	1,528,869	23,793	3.06	1.17 (1.16–1.19)	1.22 (1.20–1.24)	1.21 (1.19–1.22)
±5%	7,341,936	72,819	1.91	1 (reference)	1 (reference)	1 (reference)
≥+5%	2,213,878	16,979	1.49	1.10 (1.08–1.12)	1.07 (1.05–1.08)	1.06 (1.05–1.08)
Male						
<-5%	736,584	12,290	3.26	1.18 (1.16–1.20)	1.21 (1.19–1.24)	1.20 (1.18–1.23)
±5%	4,113,154	43,068	1.99	1 (reference)	1 (reference)	1 (reference)
≥+5%	1,246,420	9,622	1.49	1.09 (1.07–1.11)	1.06 (1.04–1.09)	1.06 (1.04–1.09)
Female						
<-5%	792,285	11,503	2.87	1.16 (1.14–1.19)	1.23 (1.20–1.26)	1.21 (1.18–1.24)
±5%	3,228,782	29,751	1.79	1 (reference)	1 (reference)	1 (reference)
≥+5%	967,458	7,357	1.50	1.11 (1.09–1.14)	1.07 (1.04–1.10)	1.07 (1.04–1.10)
P for interaction by sex				0.58	0.6101	0.7741

All data met P <0.0001. Model 1 was adjusted for age and sex; Model 2 was adjusted for the variables in Model 1 plus body mass index, smoking, alcohol drinking, regular physical activity, low-income status; Model 3 was adjusted for the variables in Model 2 plus IHD, COPD, and CKD. IR, incidence rate; HRs, hazard ratios; CIs, confidence intervals; IHD, ischemic heart disease; COPD, chronic obstructive pulmonary disease; CKD, chronic kidney disease

The results according to sex were similar for the entire study group (Table 2). In subgroup analyses that could affect the incidence of ischemic stroke, age of 60 years or older, presence of ischemic heart disease, smoking status and physical activity showed interaction with the association between weight change and ischemic stroke (S2 Table).

**Fig 2:**
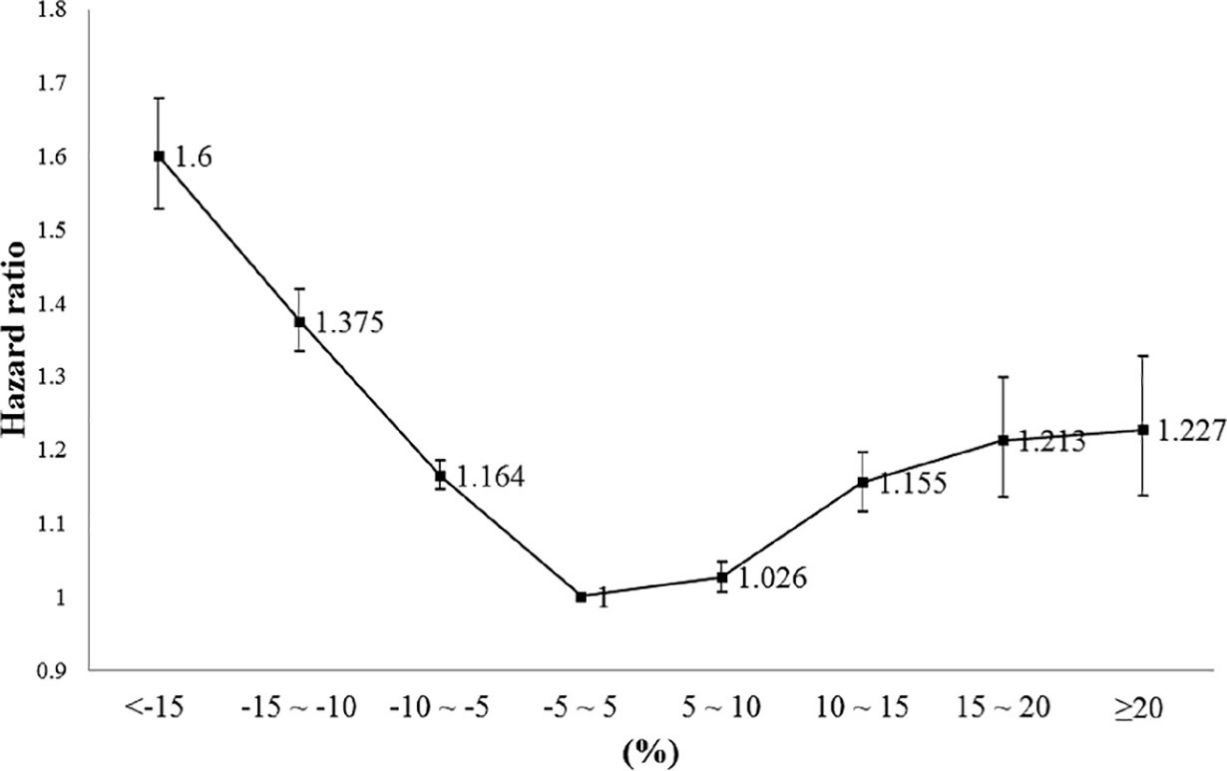
Multivariate-adjusted HR of ischemic stroke (in eight groups) using Model 3 (95% confidence intervals) in the entire study population.

After the analyses of the eight groups divided by 5% intervals (Fig 2), the risk showed a U-shaped curve with those who maintained their weight within ±5% showing the lowest risk; the risk gradually increased as weight was lost or gained in the entire population.

When the analyses were performed separately according to ICD-10 codes I63 or I64, similar trends were observed, although absolute risk was higher in those with I63 than in those with I64 due to differences in the absolute number of events (S3 Table).

When similar analyses were performed in subgroups stratified by the presence and absence of three diseases that could affect weight (chronic obstructive pulmonary disease, ischemic heart disease, and chronic kidney disease), the results were similar (S4 Table).

### Comparison of the risk for ischemic stroke according to the interval changes in BMI

The risk was analyzed according to the changes in BMI between baseline and four years prior to baseline with the reference group being those who maintained their BMI level (Table 3). The proportion of participants in low BMI levels decreased and those in high BMI levels increased after four years (S5 Table). Regardless of BMI levels four years prior to the baseline, the lowest risk was always observed in the group who maintained BMI after adjusting for all variables. The only exceptions were those with a BMI <18.5 kg/m^2^ and post 25.0–29.9 kg/m^2^ four years prior to baseline (Table 3).

**Table 3:** Comparison of the multivariable adjusted HRs (95% CIs) for ischemic stroke according to the difference between baseline- and 4-years-prior-to-baseline-BMI levels 4-years-prior-to-baseline-BMI level.

	Baseline-BMI level	Frequency	Number of events	IRs (per 1,000 person-years)	Multivariable-adjusted HRs (95% CI)
1					
	1	226,541	1,633	1.42	1 (reference)
	2	163,945	1,005	1.21	1.06 (0.98–1.15)
	3	2,307	39	3.36	1.61 (1.17–2.21)
	4	973	12	2.38	1.02 (0.58–1.80)
	5	92	2	4.26	2.05 (0.51–8.20)
2					
	1	133,715	1484	2.21	0.97 (0.92–1.02)
	2	3,409,278	28,486	1.61	1 (reference)
	3	765,201	6,362	1.60	1.08 (1.05–1.11)
	4	99,944	827	1.63	1.33 (1.24–1.42)
	5	1,961	31	3.10	1.59 (1.12–2.26)
3					
	1	1,816	52	5.93	1.20 (0.92–1.58)
	2	561,342	7,373	2.54	1.07 (1.04–1.10)
	3	1,556,689	16,185	1.99	1 (reference)
	4	683,507	6,581	1.86	1.11 (1.08–1.15)
	5	4,419	54	2.47	2.06 (1.58–2.70)
4					
	1	835	25	6.02	1.26 (0.85–1.86)
	2	62,737	1,214	3.838	1.21 (1.15–1.29)
	3	516,600	7,300	2.731	1.01 (0.99–1.04)
	4	2,426,687	29,393	2.333	1 (reference)
	5	146,039	1,412	1.908	1.11 (1.05–1.17)
5					
	1	92	3	6.714	1.57 (0.51–4.86)
	2	1,275	23	3.627	1.11 (0.74–1.68)
	3	2,549	31	2.425	0.97 (0.68–1.38)
	4	92,780	1,411	3.002	1.07 (1.00–1.14)
	5	223,359	2,653	2.349	1 (reference)

All data met P <0.0001. BMI level 1, BMI <18.5; BMI level 2, 18.5≤ BMI <23.0; BMI level 3, 23.0≤ BMI <25.0; BMI level 4, 25.0≤ BMI <30.0; BMI level 5, BMI ≥30.0. Multivariable adjustment was performed for age, sex, smoking, alcohol drinking, regular physical activity, low-income status, IHD, COPD, and CKD.

P for interaction by pre BMI * post BMI <0.0001

IR, incidence rate; HRs, hazard ratios; CIs, confidence intervals; IHD, ischemic heart disease; COPD, chronic obstructive pulmonary disease; CKD, chronic kidney disease

## Discussion

In this study of more than ten million Korean adults, nearly a quarter of the entire population, we report that the risk of ischemic stroke gradually increased in those who lost or gained more than 5% of their weight after adjustment for confounders compared to those who maintained their weight in the mean follow-up period of five years. When analyzed in eight groups of 5% intervals, the risk showed a U-shaped curve with those who maintained their body weight having the lowest risk for ischemic stroke. Our findings suggest that changes in body weight larger than 5%, whether it’s a gain or a loss, could increase the risk for ischemic stroke.

In a previous study in Asian men who were not obese on average [18], long-term weight loss from a young age had the effect of lowering the incidence of coronary heart disease (CHD), despite an increased risk for CHD in middle-aged individuals with short-term weight loss over six years. The Atherosclerosis Risk in Communities Study demonstrated the different effects of long-term (within approximately 30 years) and short-term (within three years) weight loss on cardiovascular disease (CVD) [13]. In our study which assessed a relatively short-term change in body weight over a 4-year period, the immediate risk of ischemic stroke was increased after weight loss, similar to the above mentioned results. A Japanese study of healthy Asians showed that the effect of weight loss on increasing ischemic stroke risk was lower in women than in men [14]. However, in our study, multivariable analyses that included more CVD-related covariates than the above Japanese study did not show a significant difference between men and women; both sexes had a greater risk of ischemic stroke with weight loss than with the maintenance or gain of weight. In addition, our study was designed for a homogenous population of Korean adults; therefore, bias related to sex and age-specific timing of enrollment would be less than for previous studies.

In a study that assessed CVD-related prognoses due to differences in the mechanism of weight loss, it was found that the effects of intentional and unintentional weight loss might be different [19]. Intentional weight reduction, such as decreasing calorie intake and increasing physical activity, had a positive effect on CVD risk, and the benefit increased with increased weight loss, especially in obese Caucasian women [7,20,21]. Nevertheless, whether intentional or unintentional, weight loss had a negative effect on the CVD outcome in older Caucasian men [22]. Additionally, increased CVD mortality was observed in overweight middle-aged Caucasian women with intentional weight loss, regardless of obesity-related health problems [11]. Because of our study design, we were unable to distinguish clearly between intentional and unintentional weight loss and did not get information about diet. However, in the study by Yaari et al., both voluntary and involuntary weight loss, including diet, appeared to increase CVD mortality [12]. In addition, considering that the calibration of fitness is of great importance in examining the relationship between obesity and CVD outcomes, adjusting for exercise is important in interpreting the risk [23,24]. We performed an additional adjustment involving exercise as a variable, and a subgroup analysis according to regular physical activity was also performed. The risk of ischemic stroke further increased as the weight decreased regardless of exercise. Therefore, we suggest that weight loss could have a negative effect on the risk of ischemic stroke, independent of intentionality.

Presumably, a loss of lean body mass with frailty increased the risk of ischemic stroke in our subjects. Previously, a study showed an increase in mortality in relatively healthy overweight subjects despite intentional weight loss, indicating the adverse effects of weight loss regardless of intentionality on human health. In addition, frail health was associated with atherosclerosis and subclinical CVD risk [25,26]. In our study, the weight loss group contained a higher proportion of elderly subjects as well as underlying diseases associated with frailty, such as ischemic heart disease, chronic obstructive pulmonary disease, and chronic kidney disease. In a study by Look AHEAD researchers, two-thirds of participants with a moderate to large intentional weight loss (more than 3%) showed a partial or whole weight regain [21]. It was reported that weight regain after diet-induced intentional weight loss weakened any cardiovascular benefits obtained by weight loss [27].

Interestingly, a recent study suggested that body-weight variability serves as an independent risk factor for CVD, and the level of this risk increased with increasing body weight fluctuation [28]. Although we could not confirm the detailed individual variability in weight, these findings might be helpful in interpreting the results of our study, which were that a larger weight loss had a greater impact on the risk of ischemic stroke.

Our study has several limitations. First, diet is an important factor in assessing the intentionality of weight change, and our observational study using existing data without diet information could lower the accuracy of our findings. For decades, Korean adults have been consuming less energy compared to their increasing energy intake, and the proportion of fat intake is also increasing [29]. Bearing this in mind, diet would have contributed proportionally more to weight gain in our study population. Second, we only studied the first diagnosed ischemic stroke, not other types of stroke such as hemorrhagic or recurrent stroke. However, in Korea, most strokes (approximately 60% of the total) were ischemic in nature [30]. More than 70% of stroke patients experienced their first attacks, suggesting that primary prevention plays an important role in stroke prevention [31]. Third, there was no washout period from enrollment to the onset of ischemic stroke, and this could be a problem in establishing a causal relationship. However, most (approximately 75%, data not shown) participants identified as having had an ischemic stroke were enrolled in 2009 and 2010, and the incidence in those two years was only 10% within the entire study period. Fourth, only those with a health screening record from four years prior to the baseline were eligible for the study; therefore, selection bias cannot be ruled out. Fifth, the ischemic stroke risk was lower in the smoking subgroup than in non-smokers, contrary to what is known about smoking as a risk factor for ischemic stroke. This may be due to the high percentage of non-smokers in the weight loss group, or a misinterpretation of the bias regarding causality in the study population. Sixth, atrial fibrillation, a major risk factor for embolic stroke was not included as a covariate. Finally, many metabolic characteristics of the participants at baseline showed big differences compared to those from four years prior, although we made attempts to adjust the covariates that could confound the results in any way. As fluctuations in body weight had deleterious effects and this was affected by multiple factors, statistical adjustments for these factors cannot lessen this limitation [32]. However, the finding that large changes in body weight over a 4-year period had deleterious effects on stroke development is valuable for the prevention of stroke in this population.

To our knowledge, this is the first study worldwide that confirms the relationship between weight change and ischemic stroke based on a large, nationwide population-based cohort comprising more than ten million individuals. The risk of a first ischemic stroke was observed to be lowest in the group that maintained their weight, and it gradually increased in those who experienced a weight loss or gain of more than 5% within four years. Furthermore, weight loss had a greater impact on ischemic stroke than weight gain after adjusting for confounders. It is important to keep this increased risk of ischemic stroke in patients with weight change in mind and to identify the cause of weight change to facilitate appropriate action in clinical practice. Further follow-up studies are required to identify the long-term and casual relationships between weight change and ischemic stroke.

## Supporting information

S1 Table Incidence rate and multivariable adjusted hazard ratios (95% CIs) of ischemic stroke in subgroups of various mediators. (DOCX)

S2 Table Incidence rate and multivariable adjusted hazard ratios (95% CIs) of ischemic stroke in various subgroups. (DOCX)

S3 Table Incidence rate and multivariate adjusted HRs (95% CIs) of stroke in different definition of ICD-10 code. (DOCX)

S4 Table Incidence rate and multivariable adjusted HRs (95% CIs) of ischemic stroke according to the presence of history of the three diseases* that could affect body weight maintenance. (DOCX)

S5 Table Number of participants in each groups divided by BMI at baseline and 4 years prior to the baseline years. (DOCX)
