# Supplementary material for: Maintenance of body weight is an important determinant for the risk of ischemic stroke: A nationwide population-based cohort study
Source: PLoS One. 2019 Jan 3;14(1):e0210153. doi: 10.1371/journal.pone.0210153 (PMC6317803; doi:10.1371/journal.pone.0210153)
Supplement: S1 Table — (DOCX) [file pone.0210153.s001.docx]

**S1 Table.** Incidence rate and multivariable adjusted hazard ratios (95% CIs) of ischemic stroke in subgroups of various mediators

| Subgroup | Frequency | Number of events | IRs (per 1,000 person years) | Multivariate-adjusted HRs^*^ (95% CI) | P for interaction |
| --- | --- | --- | --- | --- | --- |
| **In 3 groups** | | | | |  |
| No diabetes |  |  |  |  | 0.0189 |
| < -5% | 1,304,874 | 16,236 | 2.432 | 1.152(1.132,1.173) |  |
| ± 5% | 6,655,403 | 54,744 | 1.577 | 1(Ref.) |  |
| ≥ +5% | 2,075,500 | 13,094 | 1.225 | 1.068(1.048,1.089) |  |
| Diabetes |  |  |  |  |  |
| < -5% | 223,995 | 7,557 | 6.843 | 1.162(1.13,1.195) |  |
| ± 5% | 686,533 | 18,075 | 5.169 | 1(Ref.) |  |
| ≥ +5% | 138,378 | 3,885 | 5.529 | 1.147(1.108,1.188) |  |
| No hypertension |  |  |  |  | <0.01 |
| < -5% | 1,043,907 | 8,948 | 1.671 | 1.207(1.178,1.237) |  |
| ± 5% | 5,241,606 | 28,862 | 1.054 | 1(Ref.) |  |
| ≥ +5% | 1,698,334 | 6,741 | 0.772 | 1.053(1.025,1.081) |  |
| Hypertension |  |  |  |  |  |
| < -5% | 484,962 | 14,845 | 6.127 | 1.173(1.151,1.196) |  |
| ± 5% | 2,100,330 | 43,957 | 4.055 | 1(Ref.) |  |
| ≥ +5% | 515,544 | 10,238 | 3.859 | 1.096(1.073,1.12) |  |
| No dyslipidemia |  |  |  |  | 0.0209 |
| < -5% | 1,203,389 | 15,955 | 2.594 | 1.194(1.172,1.216) |  |
| ± 5% | 5,776,843 | 48,191 | 1.598 | 1(Ref.) |  |
| ≥ +5% | 1,778,098 | 10,891 | 1.190 | 1.047(1.025,1.069) |  |
| Dyslipidemia |  |  |  |  |  |
| < -5% | 325,480 | 7,838 | 4.815 | 1.23(1.198,1.263) |  |
| ± 5% | 1,565,093 | 24,628 | 3.061 | 1(Ref.) |  |
| ≥ +5% | 435,780 | 6,088 | 2.726 | 1.099(1.069,1.131) |  |
| Neither of the three diseases |  |  |  |  | 0.0384 |
| < -5% | 827,819 | 5,435 | 1.275 | 1.147(1.112,1.184) |  |
| ± 5% | 4,249,594 | 18,709 | 0.842 | 1(Ref.) |  |
| ≥ +5% | 1,409,889 | 4,385 | 0.605 | 1.049(1.015,1.084) |  |
| One of the three diseases |  |  |  |  |  |
| < -5% | 701,050 | 18,358 | 5.220 | 1.196(1.176,1.217) |  |
| ± 5% | 3,092,342 | 54,110 | 3.386 | 1(Ref.) |  |
| ≥ +5% | 803,989 | 12,594 | 3.044 | 1.088(1.067,1.109) |  |
| **In 8 groups** |  |  |  |  |  |
| No diabetes |  |  |  |  | 0.0289 |
| < -15% | 62,918 | 1,157 | 3.705 | 1.416(1.334,1.502) |  |
| -15 – -10% | 207,708 | 3,132 | 2.992 | 1.29(1.244,1.338) |  |
| -10 – -5% | 1,034,248 | 11,947 | 2.248 | 1.106(1.083,1.128) |  |
| ± 5% | 6,655,403 | 54,744 | 1.577 | 1(Ref.) |  |
| +5 – +10% | 1,472,484 | 9,548 | 1.252 | 1.037(1.015,1.06) |  |
| +10 – +15% | 419,735 | 2,436 | 1.139 | 1.141(1.095,1.188) |  |
| +15 – +20% | 116,110 | 633 | 1.083 | 1.163(1.076,1.258) |  |
| ≥ +20% | 67,171 | 477 | 1.419 | 1.23(1.124,1.346) |  |
| Diabetes |  |  |  |  |  |
| < -15% | 12,776 | 643 | 10.741 | 1.468(1.355,1.591) |  |
| -15 – -10% | 39,974 | 1,491 | 7.729 | 1.224(1.16,1.292) |  |
| -10 – -5% | 171,245 | 5,423 | 6.369 | 1.124(1.09,1.159) |  |
| ± 5% | 686,533 | 18,075 | 5.169 | 1(Ref.) |  |
| +5 – +10% | 100,369 | 2,688 | 5.247 | 1.083(1.039,1.127) |  |
| +10 – +15% | 25,768 | 784 | 6.043 | 1.278(1.19,1.373) |  |
| +15 – +20% | 7,205 | 239 | 6.672 | 1.426(1.255,1.62) |  |
| ≥ +20% | 5,036 | 174 | 7.014 | 1.384(1.192,1.607) |  |
| No hypertension |  |  |  |  | 0.0003 |
| < -15% | 49,648 | 583 | 2.345 | 1.521(1.4,1.653) |  |
| -15 – -10% | 167,178 | 1,713 | 2.026 | 1.374(1.307,1.443) |  |
| -10 – -5% | 827,081 | 6,652 | 1.561 | 1.154(1.123,1.185) |  |
| ± 5% | 5,241,606 | 28,862 | 1.054 | 1(Ref.) |  |
| +5 – +10% | 1,191,997 | 4,931 | 0.799 | 1.026(0.995,1.057) |  |
| +10 – +15% | 351,368 | 1,268 | 0.709 | 1.126(1.064,1.191) |  |
| +15 – +20% | 98,644 | 327 | 0.659 | 1.155(1.036,1.288) |  |
| ≥ +20% | 56,325 | 215 | 0.763 | 1.109(0.97,1.269) |  |
| Hypertension |  |  |  |  |  |
| < -15% | 26,046 | 1,217 | 9.854 | 1.437(1.356,1.523) |  |
| -15 – -10% | 80,504 | 2,910 | 7.384 | 1.272(1.224,1.321) |  |
| -10 – -5% | 378,412 | 10,718 | 5.625 | 1.13(1.106,1.154) |  |
| ± 5% | 2,100,330 | 43,957 | 4.055 | 1(Ref.) |  |
| +5 – +10% | 380,856 | 7,305 | 3.709 | 1.045(1.02,1.072) |  |
| +10 – +15% | 94,135 | 1,952 | 4.067 | 1.202(1.149,1.258) |  |
| +15 – +20% | 24,671 | 545 | 4.384 | 1.288(1.184,1.402) |  |
| ≥ +20% | 15,882 | 436 | 5.515 | 1.41(1.283,1.55) |  |
| No dyslipidemia |  |  |  |  | 0.0614 |
| < -15% | 60,440 | 1,229 | 4.113 | 1.534(1.449,1.625) |  |
| -15 – -10% | 196,901 | 3,145 | 3.176 | 1.332(1.284,1.382) |  |
| -10 – -5% | 946,048 | 11,581 | 2.382 | 1.14(1.117,1.164) |  |
| ± 5% | 5,776,843 | 48,191 | 1.598 | 1(Ref.) |  |
| +5 – +10% | 1,260,564 | 7,909 | 1.211 | 1.016(0.993,1.041) |  |
| +10 – +15% | 362,474 | 2,045 | 1.108 | 1.125(1.076,1.175) |  |
| +15 – +20% | 99,603 | 533 | 1.063 | 1.134(1.041,1.235) |  |
| ≥ +20% | 55,457 | 404 | 1.458 | 1.168(1.059,1.288) |  |
| Dyslipidemia |  |  |  |  |  |
| < -15% | 15,254 | 571 | 7.781 | 1.536(1.412,1.671) |  |
| -15 – -10% | 50,781 | 1,478 | 5.921 | 1.362(1.291,1.437) |  |
| -10 – -5% | 259,445 | 5,789 | 4.437 | 1.182(1.148,1.216) |  |
| ± 5% | 1,565,093 | 24,628 | 3.061 | 1(Ref.) |  |
| +5 – +10% | 312,289 | 4,327 | 2.692 | 1.045(1.012,1.08) |  |
| +10 – +15% | 83,029 | 1,175 | 2.781 | 1.202(1.134,1.275) |  |
| +15 – +20% | 23,712 | 339 | 2.848 | 1.347(1.21,1.5) |  |
| ≥ +20% | 16,750 | 247 | 2.944 | 1.412(1.246,1.601) |  |
| Neither of the three diseases |  |  |  |  | 0.1072 |
| < -15% | 39,639 | 328 | 1.643 | 1.339(1.199,1.496) |  |
| -15 – -10% | 132,840 | 1,050 | 1.555 | 1.315(1.235,1.401) |  |
| -10 – -5% | 655,340 | 4,057 | 1.198 | 1.101(1.064,1.14) |  |
| ± 5% | 4,249,594 | 18,709 | 0.842 | 1(Ref.) |  |
| +5 – +10% | 990,371 | 3,218 | 0.628 | 1.023(0.986,1.062) |  |
| +10 – +15% | 294,354 | 825 | 0.551 | 1.123(1.047,1.204) |  |
| +15 – +20% | 81,408 | 203 | 0.496 | 1.117(0.973,1.283) |  |
| ≥ +20% | 43,756 | 139 | 0.636 | 1.144(0.968,1.352) |  |
| One of the three disease |  |  |  |  |  |
| < -15% | 36,055 | 1,472 | 8.529 | 1.503(1.426,1.584) |  |
| -15 – -10% | 114,842 | 3,573 | 6.328 | 1.307(1.263,1.353) |  |
| -10 – -5% | 550,153 | 13,313 | 4.790 | 1.149(1.127,1.171) |  |
| ± 5% | 3,092,342 | 54,110 | 3.386 | 1(Ref.) |  |
| +5 – +10% | 582,482 | 9,018 | 2.994 | 1.042(1.019,1.066) |  |
| +10 – +15% | 151,149 | 2,395 | 3.105 | 1.185(1.138,1.235) |  |
| +15 – +20% | 41,907 | 669 | 3.169 | 1.267(1.174,1.367) |  |
| ≥ +20% | 28,451 | 512 | 3.595 | 1.335(1.223,1.456) |  |

*Model was adjusted for age, sex, body mass index, smoking, alcohol drinking, regular physical activity, low-income status, IHD, COPD, and CKD.

IR, incidence rate; HRs, hazard ratios; CIs, confidence intervals; IHD, ischemic heart disease; COPD, chronic obstructive pulmonary disease; CKD, chronic kidney disease
