# Supplementary material for: Maintenance of body weight is an important determinant for the risk of ischemic stroke: A nationwide population-based cohort study
Source: PLoS One. 2019 Jan 3;14(1):e0210153. doi: 10.1371/journal.pone.0210153 (PMC6317803; doi:10.1371/journal.pone.0210153)
Supplement: S2 Table — (DOCX) [file pone.0210153.s002.docx]

| **S2 Table.** Incidence rate and multivariable adjusted hazard ratios (95% CIs) of ischemic stroke in various subgroups | | | | | | |  |
| --- | --- | --- | --- | --- | --- | --- | --- |
| Subgroup | Frequency | Number of events | IRs (per 1,000 person years) | Multivariate-adjusted HRs^*^ (95% CI) | | | P for interaction |
|  |  |  |  | Model 1 | Model 2 | Model 3 |  |
| **In 3 groups** | | | | | | |  |
| Age < 60 |  |  |  |  |  |  | <.0001 |
| < -5% | 984,841 | 4,493 | 0.885 | 1.213 (1.175-1.253) | 1.174 (1.136 - 1.213) | 1.254(1.214,1.296) |  |
| ± 5% | 5,437,851 | 20,670 | 0.727 | 1 (reference) | 1 (reference) | 1 (reference) |  |
| ≥ +5% | 1,846,234 | 5,114 | 0.539 | 1.055 (1.023 - 1.088) | 1.024 (0.992 - 1.056) | 1.003(0.972,1.034) |  |
| Age ≥ 60 |  |  |  |  |  |  |  |
| < -5% | 544,028 | 19,300 | 7.14 | 1.182 (1.162 - 1.202) | 1.158 (1.138 - 1.178) | 1.196(1.175,1.216) |  |
| ± 5% | 1,904,085 | 52,149 | 5.329 | 1 (reference) | 1 (reference) | 1 (reference) |  |
| ≥ +5% | 367,644 | 11,865 | 6.238 | 1.145 (1.123 - 1.168) | 1.138 (1.116 - 1.161) | 1.115(1.093,1.138) |  |
| No history of TIA |  |  |  |  |  |  | 0.786 |
| < -5% | 1,479,359 | 21,661 | 2.873 | 1.17 (1.152 - 1.189) | 1.155 (1.137 - 1.173) | 1.2(1.181,1.219) |  |
| ± 5% | 7,166,175 | 67,556 | 1.810 | 1 (reference) | 1 (reference) | 1 (reference) |  |
| ≥ +5% | 2,173,545 | 15,744 | 1.408 | 1.1 (1.081 - 1.119) | 1.092 (1.074 - 1.112) | 1.064(1.046,1.083) |  |
| History of TIA |  |  |  |  |  |  |  |
| < -5% | 49,510 | 2,132 | 8.854 | 1.208 (1.148 - 1.271) | 1.19 (1.129 - 1.254) | 1.227(1.164,1.292) |  |
| ± 5% | 175,761 | 5,263 | 5.918 | 1 (reference) | 1 (reference) | 1 (reference) |  |
| ≥ +5% | 40,333 | 1,235 | 6.041 | 1.09 (1.024 - 1.159) | 1.084 (1.019 - 1.154) | 1.06(0.996,1.128) |  |
| No IHD |  |  |  |  |  |  | 0.0494 |
| < -5% | 1,427,023 | 19,434 | 2.67 | 1.17 (1.151 - 1.189) | 1.157 (1.138 - 1.176) | 1.211(1.191,1.231) |  |
| ± 5% | 6,965,939 | 61,292 | 1.689 | 1 (reference) | 1 (reference) | 1 (reference) |  |
| ≥ +5% | 2,131,282 | 14,287 | 1.303 | 1.092 (1.073 - 1.113) | 1.083 (1.064 - 1.103) | 1.054(1.035,1.073) |  |
| IHD |  |  |  |  |  |  |  |
| < -5% | 101,846 | 4,359 | 8.715 | 1.192 (1.151 - 1.235) | 1.14 (1.099 - 1.182) | 1.173(1.131,1.216) |  |
| ± 5% | 375,997 | 11,527 | 5.985 | 1 (reference) | 1 (reference) | 1 (reference) |  |
| ≥ +5% | 82,596 | 2,692 | 6.362 | 1.149 (1.102 - 1.198) | 1.155 (1.108 - 1.205) | 1.129(1.082,1.178) |  |
| Non smoker |  |  |  |  |  |  | 0.0074 |
| < -5% | 1,193,846 | 18,523 | 3.051 | 1.158 (1.139 - 1.178) | 1.169 (1.149 - 1.189) | 1.213(1.192,1.234) |  |
| ± 5% | 5,629,882 | 56,061 | 1.915 | 1 (reference) | 1 (reference) | 1 (reference) |  |
| ≥ +5% | 1,646,731 | 13,283 | 1.568 | 1.117 (1.096 - 1.138) | 1.104 (1.083 - 1.125) | 1.076(1.056,1.097) |  |
| Current smoker |  |  |  |  |  |  |  |
| < -5% | 335,023 | 5,270 | 3.084 | 1.139 (1.103 - 1.175) | 1.132 (1.096 - 1.168) | 1.18(1.143,1.218) |  |
| ± 5% | 1,712,054 | 16,758 | 1.874 | 1 (reference) | 1 (reference) | 1 (reference) |  |
| ≥ +5% | 567,147 | 3,696 | 1.267 | 1.042 (1.005 - 1.08) | 1.049 (1.012 - 1.088) | 1.017(0.981,1.054) |  |
| No regular physical activity |  |  |  |  |  |  | 0.0304 |
| < -5% | 1,200,490 | 19,812 | 3.255 | 1.173 (1.154 - 1.193) | 1.161 (1.142 - 1.181) | 1.207(1.187,1.227) |  |
| ± 5% | 5,861,802 | 58,934 | 1.937 | 1 (reference) | 1 (reference) | 1 (reference) |  |
| ≥ +5% | 1,856,205 | 14,190 | 1.489 | 1.084 (1.065 - 1.104) | 1.085 (1.065 - 1.105) | 1.056(1.037,1.075) |  |
| Regular physical activity |  |  |  |  |  |  |  |
| < -5% | 328,379 | 3,981 | 2.353 | 1.159 (1.119 - 1.201) | 1.149 (1.108 - 1.191) | 1.193(1.151,1.237) |  |
| ± 5% | 1,480,134 | 13,885 | 1.785 | 1 (reference) | 1 (reference) | 1 (reference) |  |
| ≥ +5% | 357,673 | 2,789 | 1.499 | 1.136 (1.09 - 1.183) | 1.127 (1.082 - 1.174) | 1.1(1.056,1.146) |  |
| No abdominal obesity |  |  |  |  |  |  | 0.1404 |
| < -5% | 1,190,400 | 15,426 | 2.54 | 1.195 (1.173 - 1.218) | 1.141 (1.12 - 1.164) | 1.183(1.161,1.206) |  |
| ± 5% | 5,254,289 | 41,026 | 1.496 | 1 (reference) | 1 (reference) | 1 (reference) |  |
| ≥ +5% | 1,466,278 | 8,579 | 1.133 | 1.094 (1.068 - 1.119) | 1.106 (1.081 - 1.132) | 1.076(1.051,1.102) |  |
| Abdominal obesity |  |  |  |  |  |  |  |
| < -5% | 338,469 | 8,367 | 4.902 | 1.239 (1.21 - 1.27) | 1.183 (1.154 - 1.213) | 1.233(1.203,1.264) |  |
| ± 5% | 2,087,647 | 31,793 | 2.947 | 1 (reference) | 1 (reference) | 1 (reference) |  |
| ≥ +5% | 747,600 | 8,400 | 2.2 | 1.069 (1.044 - 1.095) | 1.074 (1.049 - 1.101) | 1.048(1.023,1.074) |  |
| Baseline BMI < 25 |  |  |  |  |  |  | 0.6103 |
| < -5% | 1,233,915 | 18,777 | 2.989 | 1.202 (1.182 - 1.223) | 1.144 (1.124 - 1.165) | 1.193(1.172,1.214) |  |
| ± 5% | 4,907,262 | 43,675 | 1.709 | 1 (reference) | 1 (reference) | 1 (reference) |  |
| ≥ +5% | 1,263,745 | 8,763 | 1.345 | 1.088 (1.063 - 1.113) | 1.102 (1.077 - 1.127) | 1.073(1.048,1.098) |  |
| Baseline BMI ≥ 25 |  |  |  |  |  |  |  |
| < -5% | 294,954 | 5,016 | 3.349 | 1.235 (1.198 - 1.273) | 1.175 (1.14 - 1.211) | 1.222(1.185,1.259) |  |
| ± 5% | 2,434,674 | 29,144 | 2.303 | 1 (reference) | 1 (reference) | 1 (reference) |  |
| ≥ +5% | 950,133 | 8,216 | 1.686 | 1.074 (1.048 - 1.101) | 1.072 (1.046 - 1.099) | 1.047(1.021,1.073) |  |
| **In 8 groups** |  |  |  |  | | |  |
| Male |  |  |  |  |  |  | 0.81 |
| < -15% | 29,164 | 742 | 5.168 | 1.445(1.343-1.554) | 1.438(1.336-1.548) | 1.414(1.314-1.522) |  |
| -15 – -10% | 107,262 | 2,171 | 4.04 | 1.311(1.255-1.369) | 1.287(1.232-1.344) | 1.268(1.213-1.325) |  |
| -10 – -5% | 600,158 | 9,377 | 3.031 | 1.138(1.113-1.164) | 1.122(1.097-1.148) | 1.118(1.092-1.143) |  |
| ± 5% | 4,113,154 | 43,068 | 1.991 | 1(reference) | 1(reference) | 1(reference) |  |
| +5 – +10% | 887,184 | 7,013 | 1.513 | 1.049(1.022-1.075) | 1.051(1.024-1.077) | 1.048(1.022-1.075) |  |
| +10 – +15% | 255,902 | 1,823 | 1.39 | 1.193(1.139-1.25) | 1.192(1.137-1.249) | 1.184(1.13-1.241) |  |
| +15 – +20% | 69,122 | 460 | 1.319 | 1.225(1.118-1.343) | 1.212(1.105-1.329) | 1.203(1.097-1.319) |  |
| ≥ +20% | 34,212 | 326 | 1.904 | 1.334(1.196-1.488) | 1.303(1.168-1.453) | 1.3(1.165-1.449) |  |
| Female |  |  |  |  |  |  |  |
| < -15% | 46,530 | 1,058 | 4.628 | 1.399(1.315-1.488) | 1.425(1.338-1.517) | 1.391(1.307-1.481) |  |
| -15 – -10% | 140,420 | 2,452 | 3.491 | 1.27(1.218-1.324) | 1.272(1.22-1.327) | 1.254(1.203-1.308) |  |
| -10 – -5% | 605,335 | 7,993 | 2.601 | 1.109(1.082-1.137) | 1.107(1.079-1.135) | 1.102(1.075-1.13) |  |
| ± 5% | 3,228,782 | 29,751 | 1.794 | 1(reference) | 1(reference) | 1(reference) |  |
| +5 – +10% | 685,669 | 5,223 | 1.491 | 1.063(1.033-1.095) | 1.05(1.02-1.082) | 1.047(1.017-1.079) |  |
| +10 – +15% | 189,601 | 1,397 | 1.461 | 1.204(1.141-1.27) | 1.166(1.105-1.23) | 1.157(1.097-1.221) |  |
| +15 – +20% | 54,193 | 412 | 1.517 | 1.339(1.215-1.476) | 1.279(1.16-1.41) | 1.264(1.147-1.393) |  |
| ≥ +20% | 37,995 | 325 | 1.712 | 1.397(1.252-1.558) | 1.307(1.171-1.458) | 1.302(1.167-1.453) |  |
| Age < 60 |  |  |  |  |  |  | <.0001 |
| < -15% | 44,521 | 207 | 0.917 | 1.576 (1.374 - 1.807) | 1.508 (1.314 - 1.73) | 1.723(1.502,1.976) |  |
| -15 – -10% | 153,308 | 739 | 0.946 | 1.427 (1.326 - 1.536) | 1.36 (1.263 - 1.464) | 1.492(1.386,1.606) |  |
| -10 – -5% | 787,012 | 3,547 | 0.872 | 1.161 (1.12 - 1.203) | 1.128 (1.088 - 1.169) | 1.196(1.154,1.24) |  |
| ± 5% | 5,437,851 | 20,670 | 0.727 | 1 (reference) | 1 (reference) | 1 (reference) |  |
| +5 – +10% | 1,293,167 | 3,775 | 0.565 | 1.015 (0.98 - 1.051) | 0.995 (0.961 - 1.031) | 0.974(0.94,1.009) |  |
| +10 – +15% | 383,924 | 965 | 0.494 | 1.159 (1.087 - 1.237) | 1.104 (1.035 - 1.178) | 1.086(1.017,1.158) |  |
| +15 – +20% | 107,917 | 236 | 0.435 | 1.243 (1.093 - 1.413) | 1.14 (1.002 - 1.297) | 1.125(0.989,1.279) |  |
| ≥ +20% | 61,226 | 138 | 0.452 | 1.328 (1.123 - 1.57) | 1.149 (0.971 - 1.359) | 1.11(0.938,1.313) |  |
| Age ≥ 60 |  |  |  |  |  |  |  |
| < -15% | 29,164 | 742 | 5.168 | 1.479 (1.407 - 1.555) | 1.431 (1.36 - 1.506) | 1.531(1.455,1.611) |  |
| -15 – -10% | 107,262 | 2,171 | 4.04 | 1.306 (1.264 - 1.35) | 1.27 (1.228 - 1.313) | 1.322(1.279,1.367) |  |
| -10 – -5% | 600,158 | 9,377 | 3.031 | 1.128 (1.107 - 1.149) | 1.111 (1.09 - 1.133) | 1.142(1.121,1.164) |  |
| ± 5% | 4,113,154 | 43,068 | 1.991 | 1 (reference) | 1 (reference) | 1 (reference) |  |
| +5 – +10% | 887,184 | 7,013 | 1.513 | 1.092 (1.067 - 1.118) | 1.089 (1.064 - 1.114) | 1.068(1.043,1.092) |  |
| +10 – +15% | 255,902 | 1,823 | 1.39 | 1.262 (1.21 - 1.317) | 1.244 (1.192 - 1.297) | 1.219(1.169,1.272) |  |
| +15 – +20% | 69,122 | 460 | 1.319 | 1.357 (1.255 - 1.467) | 1.324 (1.224 - 1.431) | 1.295(1.197,1.4) |  |
| ≥ +20% | 34,212 | 326 | 1.904 | 1.445 (1.325 - 1.577) | 1.402 (1.285 - 1.529) | 1.349(1.237,1.472) |  |
| No history of TIA |  |  |  |  |  |  | 0.5644 |
| < -15% | 72,525 | 1,604 | 4.483 | 1.429 (1.36 - 1.502) | 1.418 (1.348 - 1.491) | 1.532(1.457,1.611) |  |
| -15 – -10% | 238,655 | 4,176 | 3.489 | 1.297 (1.256 - 1.338) | 1.276 (1.236 - 1.317) | 1.338(1.296,1.381) |  |
| -10 – -5% | 1,168,179 | 15,881 | 2.654 | 1.123 (1.103 - 1.142) | 1.11 (1.091 - 1.13) | 1.148(1.128,1.169) |  |
| ± 5% | 7,166,175 | 67,556 | 1.810 | 1 (reference) | 1 (reference) | 1 (reference) |  |
| +5 – +10% | 1,543,059 | 11,386 | 1.426 | 1.057 (1.036 - 1.078) | 1.054 (1.033 - 1.075) | 1.028(1.008,1.049) |  |
| +10 – +15% | 438,111 | 2,954 | 1.324 | 1.191 (1.148 - 1.236) | 1.174 (1.131 - 1.218) | 1.143(1.101,1.186) |  |
| +15 – +20% | 121,455 | 808 | 1.322 | 1.287 (1.201 - 1.379) | 1.251 (1.168 - 1.341) | 1.214(1.132,1.301) |  |
| ≥ +20% | 70,920 | 596 | 1.680 | 1.373 (1.267 - 1.489) | 1.31 (1.208 - 1.42) | 1.241(1.145,1.346) |  |
| History of TIA |  |  |  |  |  |  |  |
| < -15% | 3,169 | 196 | 13.637 | 1.486 (1.287 - 1.715) | 1.458 (1.26 - 1.688) | 1.533(1.324,1.774) |  |
| -15 – -10% | 9,027 | 447 | 10.436 | 1.283 (1.164 - 1.414) | 1.261 (1.142 - 1.392) | 1.308(1.185,1.443) |  |
| -10 – -5% | 37,314 | 1,489 | 8.111 | 1.162 (1.096 - 1.231) | 1.148 (1.083 - 1.218) | 1.18(1.113,1.251) |  |
| ± 5% | 175,761 | 5,263 | 5.918 | 1 (reference) | 1 (reference) | 1 (reference) |  |
| +5 – +10% | 29,794 | 850 | 5.599 | 1.023 (0.952 - 1.1) | 1.02 (0.949 - 1.097) | 1(0.93,1.076) |  |
| +10 – +15% | 7,392 | 266 | 7.162 | 1.28 (1.132 - 1.448) | 1.269 (1.122 - 1.436) | 1.233(1.09,1.395) |  |
| +15 – +20% | 1,860 | 64 | 6.929 | 1.192 (0.932 - 1.525) | 1.163 (0.909 - 1.489) | 1.12(0.875,1.433) |  |
| ≥ +20% | 1,287 | 55 | 8.793 | 1.336 (1.024 - 1.743) | 1.301 (0.997 - 1.698) | 1.255(0.961,1.637) |  |
| No IHD |  |  |  |  |  |  | 0.2609 |
| < -15% | 69,726 | 1,435 | 4.162 | 1.437 (1.364 - 1.515) | 1.434 (1.36 - 1.513) | 1.575(1.494,1.661) |  |
| -15 – -10% | 229,526 | 3,685 | 3.196 | 1.28 (1.238 - 1.324) | 1.265 (1.223 - 1.308) | 1.343(1.299,1.389) |  |
| -10 – -5% | 1,127,771 | 14,314 | 2.476 | 1.125 (1.105 - 1.146) | 1.115 (1.094 - 1.135) | 1.159(1.138,1.181) |  |
| ± 5% | 6,965,939 | 61,292 | 1.689 | 1 (reference) | 1 (reference) | 1 (reference) |  |
| +5 – +10% | 1,510,780 | 10,320 | 1.32 | 1.049 (1.027 - 1.071) | 1.045 (1.023 - 1.067) | 1.018(0.997,1.04) |  |
| +10 – +15% | 431,124 | 2,701 | 1.23 | 1.188 (1.144 - 1.235) | 1.169 (1.125 - 1.215) | 1.137(1.094,1.182) |  |
| +15 – +20% | 119,612 | 732 | 1.216 | 1.276 (1.186 - 1.372) | 1.237 (1.15 - 1.33) | 1.197(1.113,1.287) |  |
| ≥ +20% | 69,766 | 534 | 1.531 | 1.343 (1.234 - 1.463) | 1.274 (1.169 - 1.387) | 1.194(1.096,1.3) |  |
| IHD |  |  |  |  |  |  |  |
| < -15% | 5,968 | 365 | 13.349 | 1.439 (1.296 - 1.598) | 1.324 (1.191 - 1.473) | 1.388(1.248,1.544) |  |
| -15 – -10% | 18,156 | 938 | 10.814 | 1.355 (1.267 - 1.449) | 1.274 (1.19 - 1.364) | 1.318(1.231,1.411) |  |
| -10 – -5% | 77,722 | 3,056 | 7.915 | 1.13 (1.085 - 1.176) | 1.091 (1.048 - 1.137) | 1.12(1.075,1.166) |  |
| ± 5% | 375,997 | 11,527 | 5.985 | 1 (reference) | 1 (reference) | 1 (reference) |  |
| +5 – +10% | 62,073 | 1,916 | 5.996 | 1.092 (1.04 - 1.146) | 1.1 (1.048 - 1.155) | 1.076(1.025,1.13) |  |
| +10 – +15% | 14,379 | 519 | 7.117 | 1.275 (1.168 - 1.392) | 1.274 (1.167 - 1.391) | 1.242(1.137,1.356) |  |
| +15 – +20% | 3,703 | 140 | 7.552 | 1.323 (1.12 - 1.563) | 1.311 (1.11 - 1.549) | 1.274(1.079,1.506) |  |
| ≥ +20% | 2,441 | 117 | 9.612 | 1.552 (1.294 - 1.862) | 1.542 (1.285 - 1.851) | 1.498(1.248,1.798) |  |
| Non smoker |  |  |  |  |  |  | 0.0156 |
| < -15% | 61,458 | 1,478 | 4.897 | 1.406 (1.335 - 1.481) | 1.449 (1.374 - 1.527) | 1.558(1.478,1.642) |  |
| -15 – -10% | 195,866 | 3,684 | 3.756 | 1.287 (1.244 - 1.331) | 1.307 (1.263 - 1.352) | 1.368(1.322,1.415) |  |
| -10 – -5% | 936,522 | 13,361 | 2.791 | 1.108 (1.087 - 1.129) | 1.118 (1.096 - 1.139) | 1.154(1.132,1.177) |  |
| ± 5% | 5,629,882 | 56,061 | 1.915 | 1 (reference) | 1 (reference) | 1 (reference) |  |
| +5 – +10% | 1,177,587 | 9,527 | 1.564 | 1.068 (1.045 - 1.092) | 1.059 (1.036 - 1.082) | 1.035(1.012,1.057) |  |
| +10 – +15% | 324,792 | 2,524 | 1.525 | 1.22 (1.173 - 1.27) | 1.196 (1.149 - 1.245) | 1.165(1.12,1.213) |  |
| +15 – +20% | 89,229 | 699 | 1.553 | 1.323 (1.228 - 1.426) | 1.284 (1.191 - 1.383) | 1.245(1.155,1.342) |  |
| ≥ +20% | 55,123 | 533 | 1.927 | 1.406 (1.291 - 1.531) | 1.342 (1.232 - 1.462) | 1.275(1.17,1.388) |  |
| Current smoker |  |  |  |  |  |  |  |
| < -15% | 14,236 | 322 | 4.578 | 1.369 (1.226 - 1.529) | 1.358 (1.215 - 1.519) | 1.479(1.323,1.654) |  |
| -15 – -10% | 51,816 | 939 | 3.627 | 1.183 (1.108 - 1.264) | 1.181 (1.105 - 1.263) | 1.243(1.163,1.329) |  |
| -10 – -5% | 268,971 | 4,009 | 2.906 | 1.114 (1.076 - 1.153) | 1.108 (1.07 - 1.148) | 1.15(1.11,1.191) |  |
| ± 5% | 1,712,054 | 16,758 | 1.874 | 1 (reference) | 1 (reference) | 1 (reference) |  |
| +5 – +10% | 395,266 | 2,709 | 1.323 | 1.013 (0.973 - 1.055) | 1.022 (0.981 - 1.064) | 0.993(0.953,1.034) |  |
| +10 – +15% | 120,711 | 696 | 1.135 | 1.124 (1.042 - 1.213) | 1.13 (1.048 - 1.22) | 1.093(1.013,1.179) |  |
| +15 – +20% | 34,086 | 173 | 1.015 | 1.109 (0.955 - 1.288) | 1.106 (0.952 - 1.285) | 1.065(0.917,1.238) |  |
| ≥ +20% | 17,084 | 118 | 1.397 | 1.185 (0.989 - 1.42) | 1.167 (0.974 - 1.4) | 1.095(0.913,1.313) |  |
| No regular physical activity |  |  |  |  |  |  | 0.224 |
| < -15% | 61,003 | 1,610 | 5.403 | 1.43 (1.36 - 1.503) | 1.434 (1.364 - 1.508) | 1.551(1.475,1.631) |  |
| -15 – -10% | 195,578 | 3,951 | 4.052 | 1.29 (1.249 - 1.333) | 1.279 (1.238 - 1.322) | 1.34(1.297,1.385) |  |
| -10 – -5% | 943,909 | 14,251 | 2.96 | 1.124 (1.104 - 1.145) | 1.113 (1.093 - 1.134) | 1.151(1.13,1.173) |  |
| ± 5% | 5,861,802 | 58,934 | 1.937 | 1 (reference) | 1 (reference) | 1 (reference) |  |
| +5 – +10% | 1,308,191 | 10,123 | 1.499 | 1.038 (1.016 - 1.06) | 1.041 (1.019 - 1.063) | 1.015(0.994,1.037) |  |
| +10 – +15% | 379,144 | 2,747 | 1.426 | 1.183 (1.139 - 1.229) | 1.179 (1.135 - 1.225) | 1.146(1.103,1.191) |  |
| +15 – +20% | 106,171 | 754 | 1.414 | 1.26 (1.173 - 1.354) | 1.24 (1.154 - 1.332) | 1.199(1.116,1.289) |  |
| ≥ +20% | 62,699 | 566 | 1.808 | 1.351 (1.243 - 1.467) | 1.307 (1.203 - 1.42) | 1.237(1.138,1.344) |  |
| Regular physical activity |  |  |  |  |  |  |  |
| < -15% | 14,691 | 190 | 2.561 | 1.321 (1.145 - 1.525) | 1.33 (1.152 - 1.537) | 1.419(1.229,1.639) |  |
| -15 – -10% | 52,104 | 672 | 2.538 | 1.273 (1.178 - 1.376) | 1.255 (1.161 - 1.357) | 1.322(1.223,1.43) |  |
| -10 – -5% | 261,584 | 3,119 | 2.305 | 1.129 (1.086 - 1.174) | 1.12 (1.077 - 1.165) | 1.159(1.114,1.205) |  |
| ± 5% | 1,480,134 | 13,885 | 1.785 | 1 (reference) | 1 (reference) | 1 (reference) |  |
| +5 – +10% | 264,662 | 2,113 | 1.526 | 1.103 (1.054 - 1.155) | 1.1 (1.05 - 1.151) | 1.075(1.027,1.126) |  |
| +10 – +15% | 66,359 | 473 | 1.385 | 1.216 (1.109 - 1.332) | 1.194 (1.089 - 1.308) | 1.164(1.062,1.276) |  |
| +15 – +20% | 17,144 | 118 | 1.355 | 1.301 (1.085 - 1.559) | 1.266 (1.056 - 1.518) | 1.234(1.03,1.48) |  |
| ≥ +20% | 9,508 | 85 | 1.774 | 1.383 (1.118 - 1.712) | 1.309 (1.057 - 1.62) | 1.235(0.998,1.53) |  |
| No abdominal obesity |  |  |  |  |  |  | 0.4268 |
| < -15% | 59,227 | 1,187 | 4.071 | 1.505 (1.42 - 1.595) | 1.396 (1.316 - 1.481) | 1.495(1.409,1.586) |  |
| -15 – -10% | 197,476 | 3,130 | 3.159 | 1.331 (1.283 - 1.381) | 1.247 (1.202 - 1.294) | 1.308(1.26,1.357) |  |
| -10 – -5% | 933,697 | 11,109 | 2.319 | 1.139 (1.116 - 1.164) | 1.099 (1.076 - 1.122) | 1.133(1.109,1.158) |  |
| ± 5% | 5,254,289 | 41,026 | 1.496 | 1 (reference) | 1 (reference) | 1 (reference) |  |
| +5 – +10% | 1,066,394 | 6,287 | 1.137 | 1.057 (1.029 - 1.085) | 1.07 (1.042 - 1.099) | 1.044(1.016,1.072) |  |
| +10 – +15% | 291,882 | 1,593 | 1.067 | 1.176 (1.119 - 1.236) | 1.185 (1.127 - 1.246) | 1.152(1.096,1.211) |  |
| +15 – +20% | 73,136 | 410 | 1.106 | 1.246 (1.13 - 1.373) | 1.25 (1.134 - 1.377) | 1.204(1.093,1.327) |  |
| ≥ +20% | 34,866 | 289 | 1.639 | 1.35 (1.202 - 1.516) | 1.344 (1.197 - 1.509) | 1.257(1.12,1.412) |  |
| Abdominal obesity |  |  |  |  |  |  |  |
| < -15% | 16,467 | 613 | 7.61 | 1.49 (1.375 - 1.614) | 1.412 (1.302 - 1.532) | 1.513(1.395,1.641) |  |
| -15 – -10% | 50,206 | 1,493 | 5.999 | 1.4 (1.329 - 1.475) | 1.318 (1.25 - 1.389) | 1.381(1.31,1.455) |  |
| -10 – -5% | 271,796 | 6,261 | 4.546 | 1.189 (1.157 - 1.222) | 1.14 (1.109 - 1.172) | 1.185(1.153,1.218) |  |
| ± 5% | 2,087,647 | 31,793 | 2.947 | 1 (reference) | 1 (reference) | 1 (reference) |  |
| +5 – +10% | 506,459 | 5,949 | 2.28 | 1.019 (0.992 - 1.048) | 1.028 (1 - 1.057) | 1.004(0.977,1.033) |  |
| +10 – +15% | 153,621 | 1,627 | 2.099 | 1.178 (1.121 - 1.239) | 1.174 (1.117 - 1.234) | 1.143(1.088,1.202) |  |
| +15 – +20% | 50,179 | 462 | 1.85 | 1.255 (1.145 - 1.376) | 1.235 (1.127 - 1.354) | 1.202(1.097,1.318) |  |
| ≥ +20% | 37,341 | 362 | 1.961 | 1.323 (1.192 - 1.467) | 1.298 (1.17 - 1.44) | 1.257(1.133,1.395) |  |
| Baseline BMI < 25 |  |  |  |  |  |  | 0.1892 |
| < -15% | 68,240 | 1,668 | 4.971 | 1.499 (1.427 - 1.575) | 1.383 (1.315 - 1.454) | 1.507(1.433,1.584) |  |
| -15 – -10% | 210,602 | 3,970 | 3.765 | 1.345 (1.302 - 1.39) | 1.26 (1.219 - 1.302) | 1.327(1.284,1.372) |  |
| -10 – -5% | 955,073 | 13,139 | 2.686 | 1.14 (1.118 - 1.163) | 1.096 (1.075 - 1.118) | 1.135(1.113,1.158) |  |
| ± 5% | 4,907,262 | 43,675 | 1.709 | 1 (reference) | 1 (reference) | 1 (reference) |  |
| +5 – +10% | 934,157 | 6,409 | 1.326 | 1.047 (1.02 - 1.074) | 1.062 (1.034 - 1.09) | 1.034(1.008,1.062) |  |
| +10 – +15% | 244,965 | 1,677 | 1.339 | 1.198 (1.141 - 1.258) | 1.205 (1.148 - 1.265) | 1.174(1.118,1.232) |  |
| +15 – +20% | 59,095 | 412 | 1.374 | 1.216 (1.104 - 1.34) | 1.221 (1.108 - 1.345) | 1.178(1.069,1.299) |  |
| ≥ +20% | 25,528 | 265 | 2.054 | 1.366 (1.21 - 1.541) | 1.363 (1.208 - 1.539) | 1.324(1.174,1.494) |  |
| Baseline BMI ≥ 25 |  |  |  |  |  |  |  |
| < -15% | 7454 | 132 | 3.602 | 1.611 (1.357 - 1.911) | 1.473 (1.241 - 1.748) | 1.564(1.318,1.856) |  |
| -15 – -10% | 37,080 | 653 | 3.523 | 1.365 (1.263 - 1.475) | 1.265 (1.17 - 1.367) | 1.328(1.229,1.436) |  |
| -10 – -5% | 250,420 | 4,231 | 3.317 | 1.209 (1.17 - 1.249) | 1.156 (1.119 - 1.194) | 1.199(1.16,1.238) |  |
| ± 5% | 2,434,674 | 29,144 | 2.303 | 1 (reference) | 1 (reference) | 1 (reference) |  |
| +5 – +10% | 638,696 | 5,827 | 1.764 | 1.03 (1.002 - 1.06) | 1.034 (1.005 - 1.064) | 1.012(0.984,1.041) |  |
| +10 – +15% | 200,538 | 1,543 | 1.519 | 1.156 (1.098 - 1.216) | 1.146 (1.089 - 1.206) | 1.116(1.06,1.174) |  |
| +15 – +20% | 64,220 | 460 | 1.435 | 1.279 (1.167 - 1.403) | 1.251 (1.14 - 1.371) | 1.218(1.11,1.335) |  |
| ≥ +20% | 46,679 | 386 | 1.664 | 1.285 (1.162 - 1.42) | 1.247 (1.127 - 1.379) | 1.178(1.065,1.303) |  |
| IR, incidence rate; HRs, hazard ratios; CIs, confidence intervals; BMI, body mass index; TIA, transient ischemic attack; IHD, ischemic heart disease; COPD, chronic obstructive pulmonary disease; CKD, chronic kidney disease.  Model 1 was adjusted for age and sex;  Model 2 was adjusted for the variables in model 1 plus body mass index, smoking, alcohol drinking, regular physical activity, low-income status;  Model 3 was adjusted for the variables in model 2 plus IHD, COPD, and CKD. | | | | | | |  |
