# Supplementary material for: Maintenance of body weight is an important determinant for the risk of ischemic stroke: A nationwide population-based cohort study
Source: PLoS One. 2019 Jan 3;14(1):e0210153. doi: 10.1371/journal.pone.0210153 (PMC6317803; doi:10.1371/journal.pone.0210153)
Supplement: S3 Table — (DOCX) [file pone.0210153.s003.docx]

**S3 Table.** Incidence rate and multivariate adjusted HRs (95% CIs) of stroke in different definition of ICD-10 code*

| BMI Subgroups | Frequency | Number of events | IRs (per 1,000 person years) | Multivariate-adjusted HRs (95% CI) | | |
| --- | --- | --- | --- | --- | --- | --- |
|  |  |  |  | Model 1 | Model 2 | Model 3 |
| **In 3 groups** | | | | | | |
| I63 | | | | | | |
| < -5% | 1,529,437 | 22,765 | 2.925 | 1.175 (1.157-1.192) | 1.223(1.204,1.242) | 1.206(1.188,1.225) |
| ± 5% | 7,343,442 | 69,475 | 1.817 | 1 (Ref.) | 1(Ref.) | 1(Ref.) |
| ≥ +5% | 2,214,283 | 16,154 | 1.418 | 1.098 (1.079-1.117) | 1.063(1.044,1.081) | 1.061(1.043,1.08) |
| I64 | | | | | | |
| < -5% | 1,541,724 | 1,578 | 0.200 | 1.201 (1.133-1.271) | 1.249(1.178,1.324) | 1.228(1.158,1.302) |
| ± 5% | 7,375,040 | 4,809 | 0.125 | 1 (Ref.) | 1(Ref.) | 1(Ref.) |
| ≥ +5% | 2,222,744 | 1,197 | 0.104 | 1.148 (1.077-1.223) | 1.115(1.046,1.188) | 1.111(1.042,1.184) |
| **In 8 groups** | | | | | | |
| I63 | | | | | | |
| < -15% | 75,747 | 1,709 | 4.587 | 1.421 (1.353-1.49) | 1.585(1.509,1.664) | 1.526(1.453,1.602) |
| -15 – -10% | 247,801 | 4,431 | 3.571 | 1.298 (1.259-1.338) | 1.376(1.335,1.42) | 1.342(1.301,1.384) |
| -10 – -5% | 1,205,889 | 16,625 | 2.694 | 1.128 (1.109-1.147) | 1.165(1.146,1.186) | 1.155(1.135,1.175) |
| ± 5% | 7,343,442 | 69,475 | 1.817 | 1 (Ref.) | 1(Ref.) | 1(Ref.) |
| +5 – +10% | 1,573,122 | 11,642 | 1.430 | 1.053 (1.033-1.074) | 1.024(1.004,1.045) | 1.024(1.004,1.044) |
| +10 – +15% | 445,595 | 3,062 | 1.350 | 1.196 (1.153-1.24) | 1.152(1.111,1.194) | 1.146(1.105,1.189) |
| +15 – +20% | 123,337 | 825 | 1.329 | 1.27 (1.185-1.359) | 1.203(1.123,1.289) | 1.195(1.116,1.28) |
| ≥ +20% | 72,229 | 625 | 1.731 | 1.376 (1.271-1.488) | 1.231(1.138,1.332) | 1.241(1.147,1.343) |
| I64 | | | | | | |
| < -15% | 77,028 | 135 | 0.354 | 1.675 (1.403-1.979) | 1.853(1.557,2.205) | 1.769(1.486,2.105) |
| -15 – -10% | 250,446 | 306 | 0.242 | 1.327 (1.179-1.488) | 1.408(1.252,1.583) | 1.363(1.212,1.533) |
| -10 – -5% | 1,214,250 | 1,137 | 0.182 | 1.135 (1.063-1.211) | 1.175(1.1,1.254) | 1.161(1.087,1.24) |
| ± 5% | 7,375,040 | 4,809 | 0.125 | 1 (Ref.) | 1(Ref.) | 1(Ref.) |
| +5 – +10% | 1,578,993 | 868 | 0.106 | 1.109 (1.031-1.192) | 1.081(1.005,1.162) | 1.079(1.003,1.16) |
| +10 – +15% | 447,292 | 226 | 0.099 | 1.239 (1.081-1.412) | 1.196(1.046,1.367) | 1.185(1.037,1.355) |
| +15 – +20% | 123,844 | 63 | 0.101 | 1.364 (1.053-1.732) | 1.299(1.013,1.666) | 1.282(1,1.644) |
| ≥ +20% | 72,615 | 40 | 0.110 | 1.259 (0.907-1.693) | 1.146(0.839,1.566) | 1.15(0.842,1.571) |

All data met *P* value < 0.0001.

Model 1 was adjusted for age and sex;

Model 2 was adjusted for the variables in model 1 plus body mass index, smoking, alcohol drinking, regular physical activity, low-income status;

Model 3 was adjusted for the variables in model 2 plus IHD, COPD, and CKD.

BMI, body mass index; IR, incidence rate; ICD-10, 10^th^ International Statistical Classification of Diseases and Related Health Problems; HRs, hazard ratios; CIs, confidence intervals.

*ICD-10: I64 – ischemic stroke / I63-undetermined stroke
