# Supplementary material for: Maintenance of body weight is an important determinant for the risk of ischemic stroke: A nationwide population-based cohort study
Source: PLoS One. 2019 Jan 3;14(1):e0210153. doi: 10.1371/journal.pone.0210153 (PMC6317803; doi:10.1371/journal.pone.0210153)
Supplement: S4 Table — (DOCX) [file pone.0210153.s004.docx]

**S4 Table.** Incidence rate and multivariable adjusted HRs (95% CIs) of ischemic stroke according to the presence of history of the three diseases* that could affect body weight maintenance

| BMI Subgroups | Frequency | Number of events | IRs (per 1,000 person years) | Multivariate-adjusted HRs (95% CI) | | |
| --- | --- | --- | --- | --- | --- | --- |
|  |  |  |  | Model 1 | Model 2 | Model 3 |
| **In 3 groups** | | | | | | |
| Neither of the three diseases | | | | | | |
| < -5% | 1,242,653 | 13,904 | 2.191 | 1.159 (1.137-1.181) | 1.205(1.181,1.228) | 1.205(1.181,1.228) |
| ± 5% | 6,201,084 | 46,699 | 1.449 | 1 (Ref.) | 1(Ref.) | 1(Ref.) |
| ≥ +5% | 1,929,039 | 10,688 | 1.080 | 1.079 (1.056-1.102) | 1.043(1.022,1.066) | 1.043(1.022,1.066) |
| One the three diseases | | | | | | |
| < -5% | 286,216 | 9,889 | 6.898 | 1.195 (1.167-1.223) | 1.221(1.192,1.251) | 1.205(1.176,1.234) |
| ± 5% | 1,140,852 | 26,120 | 4.370 | 1 (Ref.) | 1(Ref.) | 1(Ref.) |
| ≥ +5% | 284,839 | 6,291 | 4.209 | 1.126 (1.095-1.157) | 1.101(1.071,1.132) | 1.107(1.077,1.138) |
| **In 8 groups** | | | | | | |
| Neither of the three diseases | | | | | | |
| < -15% | 58,686 | 946 | 3.236 | 1.44 (1.349-1.535) | 1.608(1.506,1.716) | 1.608(1.506,1.716) |
| -15 – -10% | 197,224 | 2,543 | 2.559 | 1.276 (1.225-1.328) | 1.35(1.296,1.406) | 1.35(1.296,1.406) |
| -10 – -5% | 986,743 | 10,415 | 2.059 | 1.115 (1.091-1.139) | 1.152(1.127,1.177) | 1.152(1.127,1.177) |
| ± 5% | 6,201,084 | 46,699 | 1.449 | 1 (Ref.) | 1(Ref.) | 1(Ref.) |
| +5 – +10% | 1,363,950 | 7,796 | 1.108 | 1.039 (1.014-1.064) | 1.01(0.986,1.035) | 1.01(0.986,1.035) |
| +10 – +15% | 392,504 | 1,983 | 0.996 | 1.162 (1.111-1.216) | 1.118(1.069,1.169) | 1.118(1.069,1.169) |
| +15 – +20% | 109,200 | 520 | 0.949 | 1.243 (1.138-1.353) | 1.173(1.076,1.28) | 1.173(1.076,1.28) |
| ≥ +20% | 63,385 | 389 | 1.231 | 1.37 (1.238-1.512) | 1.219(1.103,1.347) | 1.219(1.103,1.347) |
| One of the three diseases | | | | | | |
| < -15% | 17,008 | 854 | 10.703 | 1.414 (1.319-1.513) | 1.502(1.401,1.61) | 1.457(1.359,1.562) |
| -15 – -10% | 50,458 | 2,080 | 8.456 | 1.31 (1.253-1.37) | 1.354(1.293,1.417) | 1.327(1.267,1.389) |
| -10 – -5% | 218,750 | 6,955 | 6.279 | 1.145 (1.115-1.176) | 1.166(1.135,1.198) | 1.155(1.124,1.186) |
| ± 5% | 1,140,852 | 26,120 | 4.371 | 1 (Ref.) | 1(Ref.) | 1(Ref.) |
| +5 – +10% | 208,903 | 4,440 | 4.034 | 1.075 (1.041-1.109) | 1.054(1.021,1.088) | 1.059(1.026,1.093) |
| +10 – +15% | 52,999 | 1,237 | 4.479 | 1.244 (1.174-1.316) | 1.213(1.145,1.284) | 1.219(1.151,1.291) |
| +15 – +20% | 14,115 | 352 | 4.841 | 1.309 (1.176-1.452) | 1.264(1.138,1.404) | 1.275(1.147,1.416) |
| ≥ +20% | 8,822 | 262 | 5.812 | 1.357 (1.199-1.529) | 1.267(1.122,1.432) | 1.292(1.143,1.459) |

* Chronic obstructive pulmonary disease, Ischemic heart disease and chronic kidney disease

All data met P value < 0.0001.

Model 1 was adjusted for age and sex;

Model 2 was adjusted for the variables in model 1 plus body mass index, smoking, alcohol drinking, regular physical activity, low-income status,

Model 3 was adjusted for the variables in model 2 plus IHD, COPD, and CKD.

BMI, body mass index; IR, incidence rate; HRs, hazard ratios; CIs, confidence intervals.
