# Supplementary material for: Maintenance of body weight is an important determinant for the risk of ischemic stroke: A nationwide population-based cohort study
Source: PLoS One. 2019 Jan 3;14(1):e0210153. doi: 10.1371/journal.pone.0210153 (PMC6317803; doi:10.1371/journal.pone.0210153)
Supplement: S5 Table — (DOCX) [file pone.0210153.s005.docx]

**S5 Table.** Number of participants in each groups divided by BMI at baseline and 4 years prior to the baseline years

| BMI levels* | Number of those who were in each BMI level in 4 years prior to the baseline (%) | Number of those who were in each BMI level at baseline (%) |
| --- | --- | --- |
| Level 1 | 393,858 (3.6) | 362,999 (3.3) |
| Level 2 | 4,410,099 (39.8) | 4,198,577 (37.9) |
| Level 3 | 2,807,773 (25.3) | 2,843,346 (25.7) |
| Level 4 | 3,152,898 (28.4) | 3,303,891 (29.8) |
| Level 5 | 320,055 (2.9) | 375,870 (3.4) |

BMI, body mass index

*BMI level 1, BMI < 18.5 kg/m^2^; BMI level 2, 18.5 ≤ BMI < 23.0 kg/m^2^; BMI level 3, 23.0 ≤ BMI < 25.0 kg/m^2^; BMI level 4, 25.0 ≤ BMI < 30.0 kg/m^2^; BMI level 5, BMI ≥ 30.0 kg/m^2^
